# Similarity of sputum mediator signatures between e-cigarette users and COPD depends on GOLD stage and type of e-cigarette: a pilot study

**DOI:** 10.1371/journal.pone.0343940

**Published:** 2026-03-09

**Authors:** Elise Hickman, William Dabbs, Heather Wells, R. Graham Barr, Prescott Woodruff, Jill Ohar, Fernando J. Martinez, Russell Bowler, Christopher B. Cooper, Jeffrey L. Curtis, J. Michael Wells, Wassim W. Labaki, Ilona Jaspers, Julia E. Rager, Neil E. Alexis

**Affiliations:** 1 Curriculum in Toxicology and Environmental Medicine, UNC Chapel Hill, Chapel Hill, North Carolina, United States of America; 2 Center for Environmental Medicine, Asthma, and Lung Biology, UNC Chapel Hill, Chapel Hill, North Carolina, United States of America; 3 Department of Environmental Sciences and Engineering, Gillings School of Global Public Health, UNC Chapel Hill, Chapel Hill, North Carolina, United States of America; 4 Department of Medicine, Columbia University Medical Center, New York, New York, United States of America; 5 Division of Pulmonary and Critical Care Medicine, Department of Medicine and Cardiovascular Research Institute, University of California, San Francisco, California, United States of America; 6 Pulmonary, Critical Care, Allergy, Immunologic Diseases Section, Wake Forest University, Winston-Salem, North Carolina, United States of America; 7 Department of Medicine, Weill Cornell Medical Center, New York, New York, United States of America; 8 Division of Pulmonary, Critical Care and Sleep Medicine, National Jewish Health, Denver, Colorado, United States of America; 9 Departments of Medicine and Physiology, David Geffen School of Medicine, University of California, Los Angeles, California, United States of America; 10 Division of Pulmonary and Critical Care Medicine, University of Michigan, Ann Arbor, Michigan, United States of America; 11 Medical Service, VA Ann Arbor Healthcare System, Ann Arbor, Michigan, United States of America; 12 Division of Pulmonary, Allergy, and Critical Care Medicine, University of Alabama at Birmingham, Birmingham, Alabama, United States of America; University of Pittsburgh, UNITED STATES OF AMERICA

## Abstract

There is overlap in symptoms and airway pathobiology between COPD and e-cigarette (e-cig) users. We sought to determine if young adult e-cig users have similar sputum soluble mediator profiles to COPD and if this is related to the Global Initiative for Chronic Obstructive Lung Disease (GOLD) stage and generation of e-cig device. Experimental groups (n = 20–30/group) included non-smokers, smokers at risk of COPD (“pre-COPD”), mild/moderate COPD (GOLD 1/2), and severe COPD (GOLD 3) from the SPIROMICS cohort and healthy e-cig users of 3^rd^ and 4^th^ generation devices (previously published). Sputum soluble mediator profiles were compared between COPD GOLD stages and then between e-cig users of both generation devices and COPD participants by GOLD stage using a suite of computational approaches, including correlation analyses, unsupervised machine learning, and multivariate distance metrics. Inflammatory mediators were significantly increased in pre-COPD and GOLD 3 versus non-smokers and GOLD 1/2. Soluble mediator profiles of e-cig users showed patterns of overlap with COPD that were GOLD stage specific and based on shared biological functions that included proteases (MMP9, MMP2) and elastases (neutrophil elastase, myeloperoxidase). These findings indicate similarities in soluble mediator profiles between e-cig users and patients with COPD, highlighting potentially similar biological mechanisms relating to inflammation and tissue remodeling. Future studies with younger COPD cohorts, and those with preserved ratio impaired spirometry (PRISm) or bronchitis versus apical emphysema are needed to fully understand the extent of biological mechanisms that are shared between e-cig users and COPD. This pilot study represents a first step in understanding potential similarities between mediator changes in the airways of COPD patients and otherwise healthy young adult e-cig users.

## Introduction

Chronic obstructive pulmonary disease (COPD) is a leading cause of death in the United States [1]. Physical symptoms of COPD include shortness of breath, chronic cough, wheezing, and frequent respiratory infections [2]. COPD is defined by airflow obstruction that is not fully reversible (post-bronchodilator FEV1/FVC ratio < 0.7) using spirometry and is characterized by altered airway and blood inflammatory cytokines, such as elevated CRP (C-reactive protein), tumor necrosis factor-α, IL-6, IL-8, fibrinogen, as well as increased airway neutrophil numbers and activation [3–5]. It is currently not well understood how inflammatory biomarkers change with COPD disease severity and progression.

In industrialized nations, cigarette smoking is the primary risk factor for COPD [6]. Toxic substances contained in the smoke drive pathogenic processes through various mechanisms including oxidative stress, altered airway inflammatory cells, exaggerated structural cell senescence, and mitochondrial-derived necroptosis [7]. Although cigarette smoking has decreased in the United States in both adults and youth in recent decades, and e-cigarette (e-cig) use has decreased in the past 5 years [8,9], e-cig use continues to be popular among teenagers and young adults, with 6% of high schoolers and 15.5% of young adults ages 21–24 reporting current e-cig use [9–11]. Several studies have now reported harmful effects in the lungs of e-cig users [12–16] and the potential for e-cigs and the e-liquids contained within them to disrupt respiratory homeostasis [17]. Importantly and likely underappreciated for their contribution to respiratory harm, is the significant technological evolution of e-cig devices beginning with early/first generation disposable cig-a-likes, to 2^nd^ generation e-cigs with a prefilled or refillable cartridge, to 3^rd^ generation tanks or mods that can be refillable, and currently to 4^th^ generation pod mods such as JUUL, that are prefilled or refillable. With each generation of e-cig device, there have been progressive changes in operation, configuration (maximum temperature, presence/absence of a wick), and contents. Differences such as presence or absence of freebase versus nicotine salts, vitamin E acetate, cannabinoids, toxic flavoring agents, and humectants are variables of concern.

The question as to whether these evolving changes have translated into increased or reduced harm is highly clinically relevant. It is well-established that e-cig use has the potential to disrupt immune and inflammatory processes in the lung [12,13,16], and some animal and in vitro studies have implicated a link between e-cig use and features of airway injury found in COPD [18]. Indeed, an increasing number of epidemiological studies have demonstrated a link between e-cig use and COPD [19–23], but it is unknown whether e-cig use results in similar inflammatory mediator profiles found in COPD. To address this knowledge gap and test the hypothesis that e-cig users may share similarities in biomarker profiles to participants with COPD, we leveraged our previously published sputum soluble mediator data from a cohort of young adult 3^rd^ and 4^th^ generation e-cig users [24], measured the same analytes in a subset of the SPIROMICS COPD cohort, and compared soluble mediator profiles between the groups using a suite of bioinformatic approaches. This pilot study represents a first step in understanding potential similarities between mediator changes in the airways of COPD patients and otherwise healthy young adult e-cig users.

## Materials and methods

### Study cohort and sample collection

#### COPD study population.

SPIROMICS is an ongoing multi-center cohort study that enrolled approximately 3000 never-smokers (<1 pack-year), and current and former smokers (≥20 pack-years) aged 40–80 years, with or without COPD, at a baseline visit between 2010–2015 [25]. The SPIROMICS protocol was approved by the IRBs at all participating institutions and participants provided informed written consent at each study visit. COPD status was based on the Global Initiative for Chronic Obstructive Lung Disease (GOLD) guidelines, which characterizes the severity of airflow obstruction based on post-bronchodilator FEV_1_/FVC < 70% [26]. This analysis comprised a subset of participants in whom an acceptable sputum sample was obtained (as defined by squamous epithelial cell percentages < 80%, and ideally < 40% when available and minimum cell viability >50%) and differential cell counts were assessed at baseline. 20–30 participants per GOLD stage were selected to parallel the number of participants per group in the previously published e-cig study detailed below [24], as this study was sufficiently powered to detect significant differences between groups in sputum soluble mediator concentrations. Because this was a pilot study, and similar previous studies had not been conducted, we did not perform a power analysis. It is important to note that e-cig use information was not collected from SPIROMICS participants. However, because e-cig use only began to gain popularity only in 2011, and a very low percentage of adults in the 40–80 year old age range were reported to be e-cig users during the recruitment time period [27,28], dual cigarette/e-cig use was not a major concern. Samples for this study were accessed August 1, 2021, and authors did not have access to information that could identify individual participants.

#### E-cig study population.

Sputum soluble mediator data for e-cig users and age-matched controls were obtained from a previously published paper [24]. E-cig use was ascertained as described in this study [24]. Briefly, participants were healthy adult non-tobacco users or daily nicotine e-cig users (at least 10–20 puffs per day) between 18–50 years old. Exclusion criteria included current symptoms of allergic rhinitis, chronic cardiorespiratory disease, immunodeficiency, bleeding disorders, current pregnancy, and FEV_1_ less than 75% predicted during the screening visit. E-cig users were also excluded if they reported using a tobacco product other than e-cigs in the past 3 months, greater than a 10 pack-year history of smoking cigarettes, or concurrent use of multiple generations of e-cigs. Participants were considered 3^rd^ generation e-cig users if they used freebase nicotine in tank or pen style e-cigs. Participants were considered 4^th^ generation e-cig users if they used nicotine salts in newer disposable or rechargeable pod style e-cigs. Some participants were also asked about their e-cig flavor, nicotine content, and use per day (puffs or mL), but data was not complete enough (n = 12 4^th^ gen e-cig users, n = 1 3^rd^ gen e-cig user) to use these variables in downstream analyses. These data are presented by individual participant in S1 Table in S1 File.

#### Induced sputum sample collection and processing.

Sputum induction and sample processing were performed as described previously [29]. The same sputum induction and processing protocols were used for both the COPD and e-cig cohorts. In brief, sputum induction (SI) was performed using nebulized hypertonic saline (3%, 4%, 5%) inhalation for three 7-minute periods. SI was not performed if baseline percent predicted FEV_1_ was less than 35% post bronchodilator. Participants with a baseline percent predicted FEV_1_ of less than 50% started with 0.9% saline, then 3% and 4%. FEV_1_ was checked at multiple times during each 7-minute inhalation period and the procedure was terminated if the subject experienced a reduction in FEV_1_ of 20% or greater from the baseline value. Efforts were made to have participants avoid scraping the mouth/throat to reduce squamous epithelial cell contamination and that samples originated from the chest and not the throat. Samples were kept on ice throughout the collection procedure. All sputum samples were processed as soon as possible following collection but no later than 2 hours following collection. Following visual inspection of the sample, portions of raw unprocessed sample were removed for analysis of mucins, microbiome composition, and viscoelastic properties, after which the sample was further processed to generate cell-free supernatants and cell pellets. Processing for supernatants and cell pellets included the use of DTT (0.1%), filtration, homogenization, and centrifugation. Hemocytometry with Trypan Blue exclusion staining was used to assess total cell counts and cell viability assessments. All supernatant samples were stored at –80° C until assayed using Mesoscale Discovery (MSD) and ELISAs, and cytospin slides were fixed (95% ethanol), stored, and shipped at room temperature to the sputum slide reading center at UNC Chapel Hill.

### Experimental procedures

#### Induced sputum soluble mediator measurement.

The concentration of soluble mediators (n = 27) was measured in cell-free induced sputum supernatants using commercially available ELISAs (R&D Systems) and Mesoscale Discovery V-Plex assay kits (S2 Table in S1 File). Soluble mediators assessed in the COPD cohort were initially selected based on whether they were determined in the Hickman study [24] to differ significantly between e-cig groups or selected by best subsets regression analysis. Additional mediators were also included that are known to be associated with COPD [30–32]. Standard curves were diluted in buffer with an equivalent concentration of DTT to diluted samples to ensure accurate interpolation of values. Soluble mediator assays for the previous Hickman study [24] with e-cig users were performed in the summer and fall of 2021, and soluble mediator assays for the COPD cohort were performed in the spring and summer of 2022. Soluble mediator concentrations were interpolated from the standard curve run on the same plate. Plates were read on the same instruments (BMG Labtech CLARIOstar for single-plex ELISAs and MESO QuickPlex SQ 120MM for Mesoscale Discovery assays) for each of the two sample sets.

#### Differential cytospin analysis.

All cytospin slides were stained with Hema 3 and read by at least 2 independent experienced readers by the SPIROMICS sputum slide reading center. All differential cell count reads were well below the maximum allowable level of 80% squamous epithelial cells (SEC), with a mean SEC (standard error) of 30.73% (± 2.3%) across all participants in this study. The minimum allowable cell viability was 50% which was exceeded in all cases.

### Data analysis

#### Data availability and coding environment.

Data input files and code used for the analysis can be found at the Ragerlab Github [33] and Dataverse [34]. All analyses were conducted using R v4.3.0 [35] and base R functions unless otherwise noted. R packages tidyverse [36], openxlsx [37], janitor [38], rstatix [39], emmeans [40], and Table 1 [41] were used throughout the analysis.

**Table 1:** Study demographics.

	Control	Pre-COPD	Mild/Moderate COPD (GOLD 1/2)	Severe COPD (GOLD 3)	Overall p-value
**N**	25	26	29	29	
**Sex, n (%)**					0.898
Male	12 (48.0%)	15 (57.7%)	15 (51.7%)	14 (48.3%)	
Female	13 (52.0%)	11 (42.3%)	14 (48.3%)	15 (51.7%)	
**Race, n (%)**					
White	13 (52.0%)	20 (76.9%)	21 (72.4%)	19 (65.5%)	0.099
Black	9 (36.0%)	6 (23.1%)	5 (17.2%)	5 (17.2%)	
Mixed/Other	3 (12.0%)	0 (0%)	1 (3.4%)	5 (17.2%)	
Missing	0 (0%)	0 (0%)	2 (6.9%)	0 (0%)	
**Hispanic, n (%)**					
No	22 (88.0%)	26 (100%)	26 (89.7%)	28 (96.6%)	0.271
Yes	3 (12.0%)	0 (0%)	3 (10.3%)	1 (3.4%)	
**Age**					
Mean (SD)	58 (± 11)	60 (± 12)	66 (± 7.2)*^,^^^^	62 (± 8.9)	0.0212
**BMI**					
Mean (SD)	29 (± 5.5)	32 (± 5.0)	28 (± 5.4)	27 (± 6.4)^^^	0.0477
**% Predicted FEV1**					
Mean (SD)	100 (± 12)	87 (± 13)*	65 (± 16)***^,^^^^^	37 (± 3.7)^###^	<0.001
Missing	1 (4.0%)	0 (0%)	0 (0%)	0 (0%)	
**Smoking Pack-Year** **History**					
Mean (SD)	0.0010 (± 0.0050)	46 (± 20)***	57 (± 25)***	56 (± 26)***	<0.001
**Current Smoker, n (%)**					
No	25 (100%)	16 (61.5%)	13 (44.8%)	17 (58.6%)	NA
Yes	0 (0%)	10 (38.5%)	15 (51.7%)	12 (41.4%)	
Missing	0 (0%)	0 (0%)	1 (3.4%)	0 (0%)	
**Urine Cotinine (ng/ml)**					
Mean (SD)	7.4 (± 37)	390 (± 550)**	720 (± 980)***	470 (± 570)***	<0.001

For continuous variables, groups were compared using the Kruskal-Wallis test with Dunn’s test for nonparametric multiple comparisons. For GOLD 1/2, N = 6 GOLD 1 and N = 23 GOLD 2. For categorical variables, groups were compared using Fisher’s Exact Test. * p < 0.05, *** p < 0.001 in comparison with the control group. ^ p < 0.05, ^^ p < 0.01 in comparison with the at-risk group, ^###^ p < 0.001 in comparison with all other groups. BMI = Body Mass Index. NA = statistics not relevant to demographic variables.

#### Data preprocessing.

Missing mediator values were imputed with GSimp, an algorithm designed for imputation of values below the limit of detection [42], because some downstream analyses were not permissive of NAs or zeros. All mediators were detected in at least 75% of the participants, and thus, no mediators were removed due to frequent non-detects.

#### Normality assessment.

Prior to statistical testing, normality of raw and log_2_-transformed data sets was assessed quantitatively using the Shapiro-Wilk test and qualitatively using visual inspection of histograms. Log_2_-transformed data were generally more normally distributed and were used for downstream analyses.

#### Comparison of between-group differences.

Significant differences in variables between groups were determined using analysis of covariance (ANCOVA), with age, sex, current smoking status, BMI, cardiovascular condition, pulmonary vascular condition, and inhaled steroid use included as covariates. An estimated marginal means (EMM) test, which computes pairwise p-values while adjusting means for influence of covariates, was used as the post-hoc test to compare differences in endpoints between participant groups. Within the EMM test, Benjamini Hochberg p-value adjustment was applied to correct for multiple hypothesis testing.

#### Analysis of correlations between mediators and similarities between correlation matrices.

Pearson correlation coefficients and associated p-values were calculated between concentrations of raw mediators within each group and plotted using the corrplot package [43]. Correlations between mediators (co-correlated mediators) were considered significant if the absolute value of the correlation coefficient was greater than 0.6 and the p-value was less than 0.01. Overlap in correlations between groups was visualized using an UpSet plot [44,45].

#### Batch correction.

Prior to clustering analyses, non-smoking healthy controls from the e-cig cohort and COPD cohort were merged into one group, and mediator concentrations across the entire dataset were adjusted to account for batch and age effects between studies using the ComBat method within the package sva [46,47]. Group was included as a known covariate, with controls from each study pooled together into one control group as a basis for cross-study batch adjustment. Batch-adjusted mediator concentrations were used for subsequent analyses.

#### Clustering of mediator concentrations in COPD and e-cig user groups.

Heatmaps were generated with the pheatmap package [48] using group means of log2-transformed, batch-adjusted mediator concentrations as input. Two approaches were used when constructing heatmaps: 1) including all mediators measured in both studies in one heatmap, and 2) making individual heatmaps for subsets of mediators with shared biological functions [49–51]. The subsets were: 1) inflammatory mediators (IL-1α, IL-1β, IL-8, TNF-α, IL-10, IL12p70, CRP), 2) chemotactic mediators (eotaxin, eotaxin-3, IL12p40, IP-10, MCP-1, MIP-1α, MIP-1β, TARC, sICAM1, sVCAM1), and 3) proteases/enzymes (MMP-2, MMP-9, MPO, NE).

#### Determination of multivariate similarity.

To determine the mathematical similarity in soluble mediator concentrations between groups in the multivariate space, we calculated the Mahalanobis distance (the distance between the multivariate means of two groups) using the HDMD package [52]. Mahalanobis distances were converted to relative similarity scores by inverting the distance and applying min-max scaling to all pairwise similarities within a mediator subset such that the most similar pair of groups had a value of 1 and the least similar pair of groups had a value of 0. This step was performed because, within each subset of mediators (all mediators, inflammatory, chemotactic, proteases/enzymes), the magnitudes of the distance values were very different due to the number of mediators included. Therefore, the unscaled values would not allow for comparison between the different subsets of mediators. To determine the statistical significance of the distance values (e.g., whether pairs were closer or farther in the multivariate space than expected at random), permutation testing was performed. Group labels were shuffled and Mahalanobis distances were recalculated (10,000 permutations) to generate a random/null distribution of distances. Actual (“real”) distance values were compared to the random distribution. Actual distance values falling in the upper 95^th^ percentile (far distance) or lower 5^th^ percentile (close distance) were considered statistically significant.

## Results

### Subject demographics

The COPD study cohort comprised a total of n = 109 participants, 25–29 per group, with no significant differences between groups in sex, race, or ethnicity (**Table 1**). Participants in GOLD stages 1/2 were significantly older than controls. Aside from non-smoking controls, each group contained a roughly equal mix of current tobacco cigarette smokers and former smokers. As expected, mean urine cotinine levels were significantly higher in all GOLD stages than controls. A few participants who did not report current smoking had measurable levels of urine cotinine. This could be the result of environmental exposure or use of vaping products. We also explored the prevalence of a wide range of comorbidities, including pulmonary vascular and cardiovascular conditions, and the prevalence of medication use (fluticasone salmeterol, oral corticosteroids, theophylline) (**S3 Table in S1 File**). Most participants did not use oral corticosteroids (n = 2 in the GOLD 3 group), and no participants used theophylline. Some participants (4–35% within each group) used fluticasone salmeterol, although there was a high amount of missing data across all groups. 32% of controls and 62–69% of participants in the pre-COPD, GOLD stages 1/2, and GOLD stage 3 groups reported a cardiovascular condition. No controls reported a pulmonary vascular condition, while 14–27% of participants within the pre-COPD, GOLD stages 1/2, and GOLD stage 3 groups did. The proportion of participants using inhaled steroids increased with disease progression, with approximately half of participants using inhaled steroids in GOLD stages 1/2 and GOLD stage 3 groups. E-cig use data for participants who completed the questionnaire (n = 12 4^th^ gen e-cig users; n = 1 3^rd^ gen e-cig user) are available in **S1 Table in S1 File**, and demographics for the previously published e-cig cohort are available in **S4 Table in S1 File**. 4^th^ gen e-cigarette users who completed the questionnaire had been using e-cigarettes for an average of 1.4 years and generally preferred mint flavor with 5% nicotine.

### Induced sputum differential cell counts

We observed no statistically significant effects of BMI, sex, race, current smoking status, or pulmonary vascular condition on differential cell count parameters (**S5 Table in S1 File**). Age was a significant covariate for neutrophil count and percentage; cardiovascular condition was a significant covariate for bronchial epithelial count, bronchial epithelial percentage, and neutrophil percentage; and inhaled steroid use was a significant covariate for eosinophil percentage (**S5 Table** in S1 File). After adjusting for these factors, sputum total cell count, neutrophil count, neutrophil percentage, and macrophage/monocyte percentage differed significantly between COPD groups (**Table 2**). In comparison with controls, total cell count, neutrophil count, and neutrophil percentage were increased, and macrophage/monocyte percentage decreased, in participants categorized as pre-COPD and GOLD stage 3, reflecting elevated inflammation in those groups. In contrast, GOLD stages 1/2 did not significantly differ from controls. Detailed statistical results per variable and pairwise comparison are presented in **S6 Table in S1 File**.

**Table 2:** Sputum cell differential counts and percentages by group.

	Control	Pre-COPD	Mild/ Moderate COPD (GOLD 1/2/)	Severe COPD (GOLD 3)	Overall p-value
**Cell Counts**					
Total Cell Count	879,484 (± 942,713)	1,807,021 (± 2,381,299)	870,452 (± 767,279)	**3,566,956 (± 6,220,882)**	**9.00E-03**
Neutrophil	283,405 (± 363,487)	**1,113,653 (± 2,229,455)**	454,829 (± 537,254)	**2,893,700 (± 5,718,846)**	**1.35E-04**
Macrophage/Monocyte	200,913 (± 224,125)	198,701 (± 234,604)	145,067 (± 138,946)	310,904 (± 412,755)	8.95E-01
Eosinophil	3,795 (± 8,189)	6,928 (± 12,260)	14,621 (± 44,224)	18,912 (± 57,766)	8.93E-01
Lymphocyte	2,204 (± 6,022)	1,150 (± 2,872)	1,349 (± 5,737)	4,011 (± 14,781)	1.63E-01
Bronchial Epithelial Cell	13,299 (± 26,946)	13,964 (± 20,927)	9,049 (± 15,232)	22,816 (± 51,951)	6.10E-02
**Cell Percentages**					
Neutrophil	52.17 (± 25.11)	**69.41 (± 22.56)**	66.24 (± 18.27)	**81.61 (± 12.19)**	**7.21E-05**
Macrophage/Monocyte	43.77 (± 22.53)	**26.36 (± 19.71)**	29.67 (± 18.81)	**14.64 (± 10.14)**	**2.38E-04**
Eosinophil	0.55 (± 1.19)	1.35 (± 2.86)	1.67 (± 3.02)	2.07 (± 5.03)	8.89E-01
Lymphocyte	0.19 (± 0.46)	0.07 (± 0.12)	0.15 (± 0.51)	0.13 (± 0.16)	8.44E-01
Bronchial Epithelial Cell	3.31 (± 5)	2.81 (± 5)	2.28 (± 3.40)	1.55 (± 2.49)	1.37E-01

Data are presented as mean (± SD). Total cell count includes squamous epithelial cells. Cell percentages were calculated using the total cell count without squamous epithelial cells. Groups were compared using ANCOVA (overall p-value) followed by an estimated margin of means test (pairwise comparisons). Benjamini-Hochberg p-adjustment was applied to correct for multiple hypothesis testing. Data in bold show significant p-values. Bolded data within each group indicate significance of at least p < 0.05 in comparison with the control group. Additional significant comparisons can be viewed in S4 and S5 Tables in S1 File.

### Differences in soluble mediators between COPD GOLD stage

**Fig 1:**
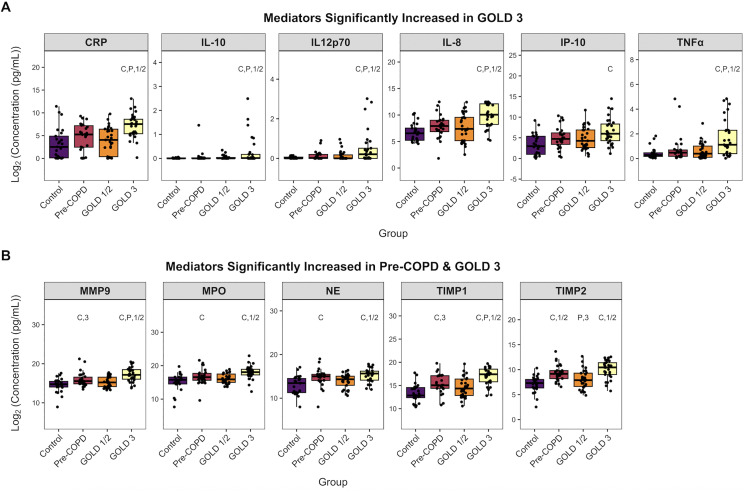
Soluble mediators that were significantly different between COPD cohort controls and GOLD stages. **(A)** Soluble mediators that were significantly increased in GOLD stage 3 vs. controls (C) after adjusting for age, sex, race, current smoking status, BMI, cardiovascular conditions, and pulmonary vascular conditions. **(B)** Soluble mediators that were significantly increased in pre-COPD (P) and GOLD stage 3 vs. controls (C) after adjusting for covariates. Results are presented as box and whisker plots of log2-transformed data, with individual data points overlaid. Annotation numbers indicate significance of at least p < 0.05 between the two groups using ANCOVA followed by estimated marginal means post-test. For example, within the CRP analysis, samples from controls **(C)**, pre-COPD **(P)**, and GOLD stages 1/2 showed significantly different expression in comparison to GOLD stage 3.

Soluble mediator concentrations by group are summarized in **S7 Table in S1 File**. We observed minimal statistically significant effects of covariates on soluble mediator concentrations (**S8 and S9 Tables in S1 File**). Forest plots for proteins with significant overall ANCOVA p-values for covariates are presented in **S1 Fig** in S2 File. Of the 27 soluble mediators measured, 14 were significantly different between groups in the adjusted analysis. Of these 14, there were statistically significant pairwise comparisons in 10. We observed two general patterns across COPD GOLD stage: (a) mediators that were significantly elevated in GOLD stage 3 only (CRP, IL-10, IL-12p70, IL-8, IP-10, and TNF-α) (**Fig 1A**); (b) mediators that were significantly elevated in both pre-COPD and GOLD stage 3 (MMP9, MPO, NE, TIMP1, TIMP2) (**Fig 1B**). Pairwise p-values reported in full for all in **S10 Table in S2 File.**

**Fig 2:**
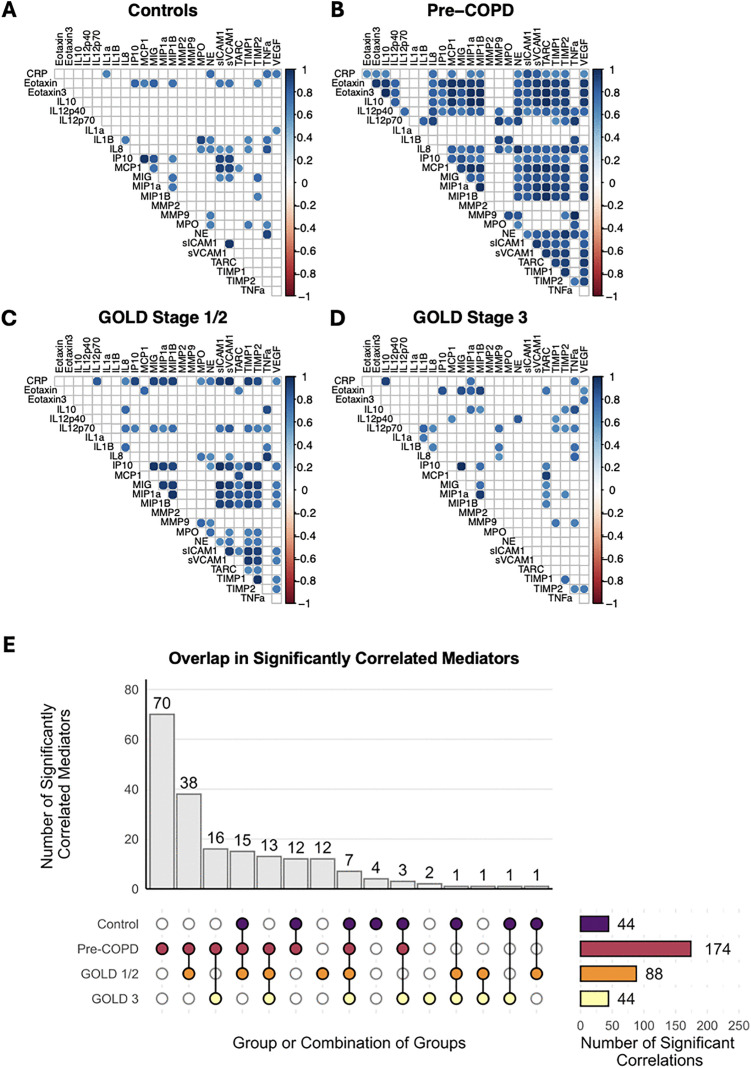
Soluble mediator correlation matrices within groups and similarities across COPD groups. **(A-D)** Correlations with Pearson correlation coefficients (|R|) > 0.6 and associated p-values < 0.01 are plotted by group. **(E)** UpSet plot showing total number per group and overlap in significantly correlated mediator pairs across groups.

To capture the overall soluble mediator milieu and degree of co-correlation between mediators (i.e., the number of mediator pairs that were correlated to each other, representing the strength of relationship between concentrations of mediators within each group), we next compared correlation matrices between GOLD stage (Fig 2A-2D). We observed that within pre-COPD participants, many soluble mediators were significantly positively correlated (n = 174 pairs of mediators), while in the control group, GOLD stages 1/2, and GOLD stage 3, there were notably fewer mediator to mediator correlations (n = 44–88 pairs of mediators). When comparing overlap of correlated mediator pairs between groups, GOLD stages 1/2 shared the most correlations with pre-COPD (n = 38 pairs of mediators), followed by GOLD stage 3 (n = 16) and control (n = 12) (**Fig 2E**). This suggests that in pre-COPD, mediator concentrations change in concert with each other and may indicate underlying protective or pathophysiological processes related to the inflammatory milieu.

### Similarities between soluble mediator profiles in COPD and e-cig use

#### Hierarchical clustering.

**Fig 3:**
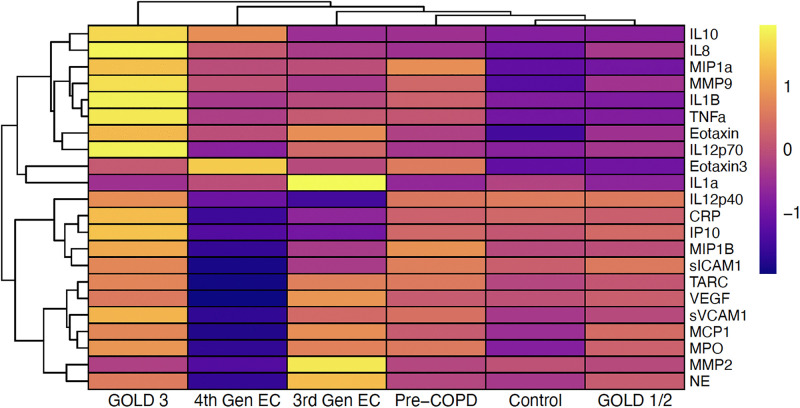
Clustering of participants with COPD and e-cig users across all soluble mediators. Row-scaled heatmap of log2, batch-adjusted mediator concentration means by group are shown. The Control group represents a combined group containing COPD study controls and non-smokers/non-e-cig users from the Hickman et al. e-cigarette study. EC = e-cig.

We next set out to understand similarities in soluble mediator concentrations across all analytes and within subsets of biological mediators that have similar functions. We employed unsupervised hierarchical clustering of all mediators, combining the never-smokers from the previous study [24] and the SPIROMICS participants into a single control group to adjust for batch and age effects. Results from clustering on mean concentrations per group demonstrated notable differences across groups, with GOLD stage 1/2 and the control group being most similar, and GOLD stage 3 being the most unique (**Fig 3**).

**Fig 4:**
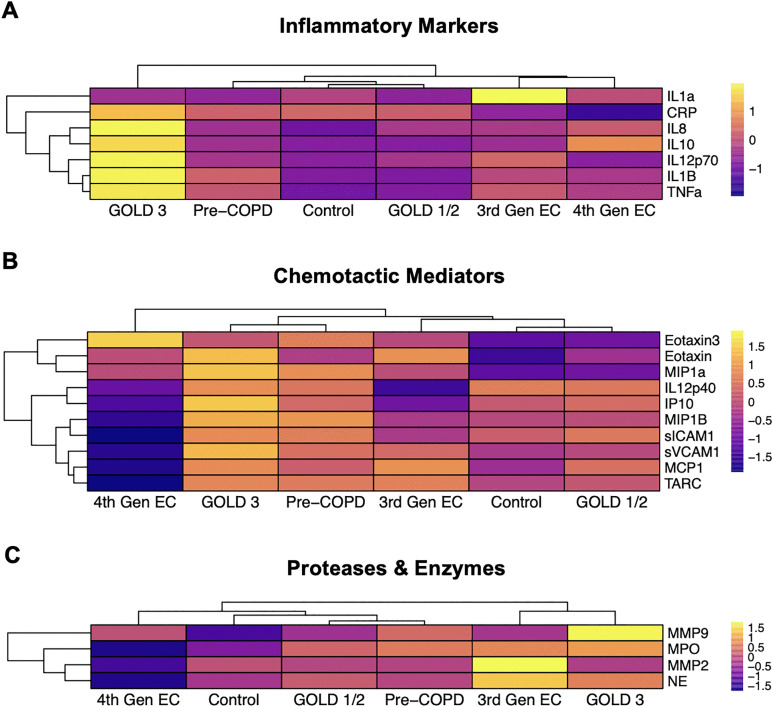
Clustering of participants with COPD and e-cig users across biological subsets of soluble mediators: (A) Inflammatory markers, (B) Chemotactic mediators, and (C) Proteases and enzymes. Row-scaled heatmaps of log2, batch-adjusted mediator concentration means by group are shown. The Control group represents a combined group containing COPD study controls and non-smokers/non-e-cig users from the Hickman et al. e-cig study. EC = e-cig.

To understand whether clustering was driven by subsets of mediators with shared biological functions, we then performed hierarchical clustering on subsets of mediators (**Fig 4**). Different patterns of clustering based on mean concentrations per group were observed depending on the subset of mediators used as input. For inflammatory markers, both e-cig use groups clustered together, while pre-COPD, GOLD stage 1/2, and controls clustered together (**Fig 4A**). GOLD stage 3 showed the most unique profile, with high expression across most of the mediators (**Fig 4A**). For chemotactic mediators, 4^th^ generation e-cig users were the most unique from other groups, with low expression across most of the mediators (**Fig 4B**). We also observed co-clustering of pre-COPD and GOLD stage 3, and GOLD stages 1/2, 3^rd^ generation e-cig users, and the composite control participants (**Fig 4B**). For the protease and enzyme subset, we observed co-clustering of 3^rd^ generation e-cig users and GOLD stage 3, with all other groups in another cluster, and 4^th^ generation e-cig users again showing lower levels than other groups (**Fig 4C**).

We also performed clustering on each individual participant’s mediator concentrations (rather than group means described above), with all mediators together or by biological function subset (**S2 and S3 Figs in S2 File**). Here, clustering results were less pronounced, as there was a high level of interindividual variability across participants, resulting in participants from different groups interspersed throughout the clusters. We observed the most apparent clustering on individual level data when assessing chemotactic mediators, as pre-COPD, GOLD 1/2, and GOLD 3 participants largely clustered with others in the same group (**Fig S3B in S2 File**).

#### Mahalanobis distance.

**Fig 5:**
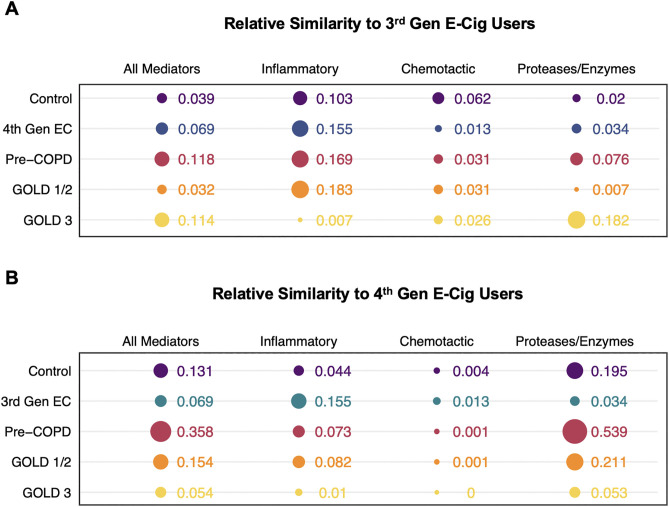
Mathematical similarity in overall and biological subset soluble mediator profiles between e-cig user groups and other groups of interest. **(A)** Relative similarity between 3^rd^ generation e-cig users and other groups, **(B)** Relative similarity between 4^th^ generation e-cig users and other groups. Similarities were derived from taking the inverse of the pairwise Mahalanobis distance and min-max scaling distances within each mediator data set (all mediators together or biological subsets) such that the most similar pair had a similarity of 1 and the least similar pair had a similarity of 0. The size of the circle is a visual representation of the magnitude of the similarity, with coloring by group. The Control group represents a combined group containing COPD study controls and non-smokers/non-e-cig users from the Hickman et al. e-cig study. EC = e-cig.

To determine the mathematical similarity between e-cig user and COPD soluble mediator profiles overall and by biological function subset, we derived a similarity score from pairwise Mahalanobis distances. The final values were scaled such that, for each anchor group, the group with the highest similarity to the anchor group had a similarity value of 1, while the group with the lowest similarity to the anchor group had a value of 0. 3^rd^ generation e-cig users were most similar to pre-COPD and GOLD stage 3 when all mediators were used as input (**Fig 5A**). When mediators were subset by biological function, 3^rd^ generation e-cig user soluble mediator profiles were most similar to pre-COPD and GOLD 1/2 for inflammatory markers and GOLD 3 for proteases and enzymes. However, 3^rd^ generation e-cig users had overall very low similarity to other groups, with the highest similarity score being 0.183 (maximum across entire dataset = 1). In contrast, 4^th^ generation e-cig users had overall higher similarity to other groups than 3^rd^ generation e-cig users, with the most similarity to pre-COPD when all mediators were included (0.358) and for the protease/enzyme subset (0.539) (**Fig 5B**). Similarity scores for each pair of groups reported in full in **Table S11 in S1 File.**

To assess whether the mathematical similarities and differences we observed between groups were more likely to be real observations than to occur by random chance, we compared the observed pairwise Mahalanobis distances to a random distribution of distances generated by permuting labels and recalculating distances (10,000 permutations). We found that for Mahalanobis distances calculated based on the dataset containing all mediators, controls and GOLD stage 1/2 participants were significantly closer to each other in multivariate space, while controls and GOLD3 participants were significantly farther apart than expected by random chance (**S4 Fig in S2 File, S12 Table in S1 File**). For immune/inflammatory mediators and chemotactic mediators, pre-COPD and GOLD stage 1/2 participants were significantly closer to each than that expected by random chance (**S5 Fig in S2 File, S12 Table in S1 File**). GOLD stage 3 participants were significantly farther apart from all other groups for immune/inflammatory mediators, and 4^th^ gen e-cig users were significantly farther apart from all other groups for chemotactic mediators (**S5 and S6 Figs in S2 File, S12 Table in S1 File**). For proteases and enzymes, controls and GOLD stage 1/2 were significantly closer to each other than expected by random chance (**S7 Fig in S2 File, S12 Table in S1 File**). Although distances between e-cigarette user groups and COPD groups did not reach statistical significance, we noted a trend towards similarity. 4^th^ gen e-cig users were close in multivariate space to pre-COPD participants for the all mediator dataset (15^th^ percentile) and the protease and enzyme dataset (11^th^ percentile), and 3^rd^ gen e-cig users were close in multivariate space to GOLD stage 3 participants in the chemotactic mediators dataset (11^th^ percentile).

## Discussion

In this pilot study, we used traditional univariate analytical approaches as well as more novel multivariate approaches, such as correlation analysis, machine learning, and distance metrics to compare sputum soluble mediator profiles by GOLD stage in COPD participants. We further assessed whether these profiles were similar to soluble mediator signatures found in young adult 3^rd^ and 4^th^ generation e-cig users, making efficient use of existing biological samples to investigate the hypothesis that e-cig users may share similarities in biomarker profiles to participants with COPD. Primary findings included the following: (1) significantly increased levels of soluble mediators in GOLD stage 3 and pre-COPD in comparison with healthy controls; (2) an increased number of co-correlated mediators in pre-COPD compared with other groups; (3) partial overlap between e-cig users’ mediator profiles and those of participants with COPD, as revealed through hierarchical clustering and distance metrics; and (4) improved clustering when mediators were separated by biological function (i.e., “expert-driven machine learning”).

We found significantly increased neutrophil levels and soluble markers of inflammation and tissue damage in pre-COPD and GOLD stage 3 as compared to healthy non-smoking controls and GOLD stages 1/2. Notably, the cytokines that were significantly increased in both pre-COPD and GOLD stage 3 (pre and late-stage disease, respectively) had biological functions specifically related to tissue damage and remodeling. These included metalloproteinases MMP9 and MMP2, neutrophil elastase, and myeloperoxidase, whereas the cytokines that were significantly increased only in GOLD stage 3 were more broadly characterized as pro-inflammatory cytokines. Although some of these markers have been previously shown to be elevated in mixed granulocytic [53,54] or neutrophilic [55] COPD or in stable versus exacerbated COPD (AECOPD) [31], the differential pattern of cytokine expression across GOLD stage reported here is a novel observation that suggests airways inflammation is a later stage developing feature whereas remodeling and tissue damage develops before and persists into later stage disease. Our correlation analysis also revealed a marked increase in the number of co-correlated cytokines in pre-COPD, potentially representing a pre-disease pattern of activity not yet present in GOLD stages 1/2 that ultimately progresses into more mature disease processes in GOLD stages 3–4.

Emerging evidence supports similarities between COPD and e-cig user phenotypes, specifically identifying shared features of lung dysfunction that include airspace enlargement, mucous cell hypertrophy, release of inflammatory mediators and altered epithelial ciliary function [18–21]. We found that, for the subset of mediators that were proteases or enzymes, 3^rd^ generation e-cig users clustered with COPD GOLD stage 3 more so than 4^th^ generation e-cig users. We also found that the Mahalanobis multivariate distance metric indicated that 4^th^ generation e-cig users were most similar to pre-COPD. When tested against random distance distributions, we found trends towards similarity between e-cig user groups (4^th^ gen e-cig and pre-COPD; 3^rd^ gen e-cig and GOLD stage 3), though these results did not reach statistical significance. Unlike univariate methods, multivariate analysis methods such as those we employed allow for integration and pattern detection across the entire mediator milieu and therefore considers the dynamic relationship between several mediators, rather than each mediator examined in isolation. This is important because airway soluble mediators often act in concert *in vivo* to achieve immune homeostasis or in the cases of disease, reveal a shift towards a disease state. Our novel multivariate analytical approach revealed a more nuanced observation, namely that similarities between 4^th^ gen e-cig users and participants with COPD were largely in the protease/enzyme cytokine subset. However, it is important to emphasize that similarities in mediator profiles do not mean that e-cig use causes the mediator profiles seen in COPD; rather, these initial observations demonstrate potentially similar biological mechanisms of disrupted immune homeostasis that differ according to GOLD stage and use of e-cig device. It is also important to acknowledge that permutation testing for statistical significance of distance metrics is limited by the endpoints and sample size measured in the present study, which may not fully capture the true random distribution of distances for the entire secreted proteome.

We hypothesize that shared mediator profiles may be in part due to shared mechanisms. In chronic airway diseases such as COPD, a protease–antiprotease imbalance has long been associated with tissue injury and disease progression. Indeed, aberrant proteolytic activity resulting from high levels of neutrophil elastase (NE) and matrix metalloproteinases (MMPs), are widely associated with episodes of acute exacerbation and pulmonary decline [56]. E-cig aerosol extract has also been shown to stimulate the release of MMP-9 and interleukin 8 from isolated neutrophils and increase NE and MMP-9 activity [57]. In humans, significantly elevated levels of NE and MMP-2/9 in e-cig users (including those who reported no prior cigarette use) as compared with nonsmokers have been observed, with no change in relevant protease inhibitors such as alpha-1 antitrypsin and tissue inhibitor of metalloproteinases-1 and −2, indicating an overall increase in proteolysis [12], Interestingly, these same authors showed that the increase in NE was independent of the humectants propylene glycol and vegetable glycerin and was associated with a rise in cytosolic calcium levels in response to nicotine. Nicotine has been associated with the progression of COPD by inducing oxygen radical production, decreasing anti-oxidant levels, and promoting pyroptosis, a unique form of inflammatory cell death, that is mediated by the activation of caspase-1 and the NOD-like receptor protein-3 (NLRP3) [58]. Animal and *in vitro* evidence also suggests a direct link between e-cig-induced lung injury and features seen in COPD that are nicotine dependent [18]. For example, mice and human bronchial epithelial cells exposed to nicotine containing, but not nicotine-free, e-cig liquid exhibited increased airway hyper-reactivity, distal airspace enlargement, mucin production, cytokine and protease expression in the former, and impaired ciliary beat frequency, airway surface liquid volume, and CFTR expression in the latter [18]. Nicotine therefore may be one important common mechanistic link between e-cig use and COPD biomarker profiles. Notably, although previous studies have demonstrated that nicotine salts in e-cigarettes produce a smoother sensory experience [59] and result in higher blood nicotine concentrations [60] than freebase nicotine, no studies to our knowledge have mechanistically evaluated the effects of freebase versus nicotine salt e-cigarettes on protease-antiprotease balance, inflammation, and oxidative stress in the respiratory tract.

Although our study is the first to demonstrate partial biomarker overlap in cytokine profiles between e-cig users of different devices and specific GOLD stages in COPD, certain limitations were present. Firstly, we were limited to interrogating soluble mediators in COPD that were also measured in the previously published e-cig user cohort. Secondly, the number of COPD participants per group was relatively low. Therefore, lack of significance for certain comparisons may be due to insufficient power rather than lack of a relationship. Thirdly, although exact information on e-cig use history (particularly frequency, dosage, and specific product type) was difficult to acquire, most e-cig users in our cohort had only been using e-cigs for a few years. In terms of former smoking, we acknowledge more detailed information is needed, but most e-cig users with a smoking history had quit smoking at least 6 months to a year prior to sample collection. Another important factor that distinguished e-cig users from COPD was the relatively younger aged e-cig cohort, although it should be noted age was not a significant modifier of soluble mediator levels and was adjusted for in the batch-corrected analysis. These limitations will be become easier to address in future studies because it will be possible to recruit older e-cig users with longer use histories as this habit continues to grow in popularity, and interrogating a more recent similarly aged population of at-risk COPD participants is of great interest. Future studies leveraging unbiased analysis methods (e.g., RNA-seq, proteomics, and metabolomics) with larger cohorts possessing equally detailed current and former smoking and e-cig use data will also be needed to further uncover shared pathophysiology between e-cig users and participants with existing respiratory disease. Inclusion of lung function assessment, high resolution CT imaging, and symptom burden analysis in future studies would also enable determination of whether changes in mediator signatures reflect clinical signs of lung disease, as the similarities described in this study are limited to mathematical similarity scoring rather than clinical similarity. It is important to acknowledge that 1) mathematical similarity is not synonymous with clinical similarity/significance; 2) mathematical similarity can exist independently from/without clinical similarity; and 3) clinical significance can exist without or not be supported by mathematical similarity, particularly if markers of mechanisms at play are not fully captured by a study. Additionally, we were not able to assess the impact of THC exposure on pathobiology because THC use information was not collected; however, there is an urgent need to assess this relationship, particularly given evidence of dual use of nicotine and THC-containing products [61]. More detailed and complete questionnaires for both THC and nicotine e-cigarette use will be necessary in future studies to investigate the impacts of use frequency and product type on soluble mediator concentrations. Importantly, although e-cig user samples were collected approximately 5 years ago, nicotine salt e-cigs (4^th^ generation) remain the most popular type of nicotine e-cig among young adults [62]. Future investigations will also benefit from a prospective study design, as the cross-sectional design in this study does not allow for causal inference.

Overall, our preliminary findings are suggestive of overlapping biology between e-cig users and individuals with spirometrically defined COPD with subtle differences according to GOLD stage and generation of e-cig device used. Importantly, we also demonstrated the application of a suite of univariate and multivariate computational approaches to uncover otherwise nuanced correlations between protein signatures that act in concert to exert effects on the airway. Overall, the limitations of this study were driven largely by its cross-sectional, preliminary nature and the integration of data from two independent studies with differing original goals. Still, data analysis methods selected for use in this study were aimed to reduce the impact of cohort-specific differences, while highlighting similarities vs differences across molecular patterns while adjusting for study-specific variation. Importantly, our results suggest that e-cig use may not be a “safe” choice for young adults. These findings lay the much-needed foundation for future prospective and more comprehensive studies will be designed to validate these findings and further examine whether or not there is a causal link between e-cig-induced airway injury and biological features of COPD, specifically examining the mechanisms underlying the mediator similarities we observed in this study.

## Supporting information

S1 File Supplementary Tables File. Supplementary tables S1-S12.
(XLSX)

S2 File Supplementary Figures File. Supplementary figures S1-S7.
(DOCX)
