# Supplementary material for: Similarity of sputum mediator signatures between e-cigarette users and COPD depends on GOLD stage and type of e-cigarette: a pilot study
Source: PLoS One. 2026 Mar 9;21(3):e0343940. doi: 10.1371/journal.pone.0343940 (PMC12970936; doi:10.1371/journal.pone.0343940)
Supplement: S2 File — Supplementary figures S1-S7. (DOCX) [file pone.0343940.s002.docx]

**Supplementary Figures:**

Similarity of Sputum Mediator Signatures Between E-Cigarette Users and COPD Depends on GOLD Stage and Type of E-Cigarette: A Pilot Study

Elise Hickman, William Dabbs, Heather Wells, R. Graham Barr, Prescott Woodruff,
Jill Ohar, Fernando J. Martinez, Russell Bowler, Christopher B. Cooper, Jeffrey L. Curtis, J Michael Wells, Wassim W. Labaki, Ilona Jaspers, Julia E. Rager , Neil E. Alexis

| **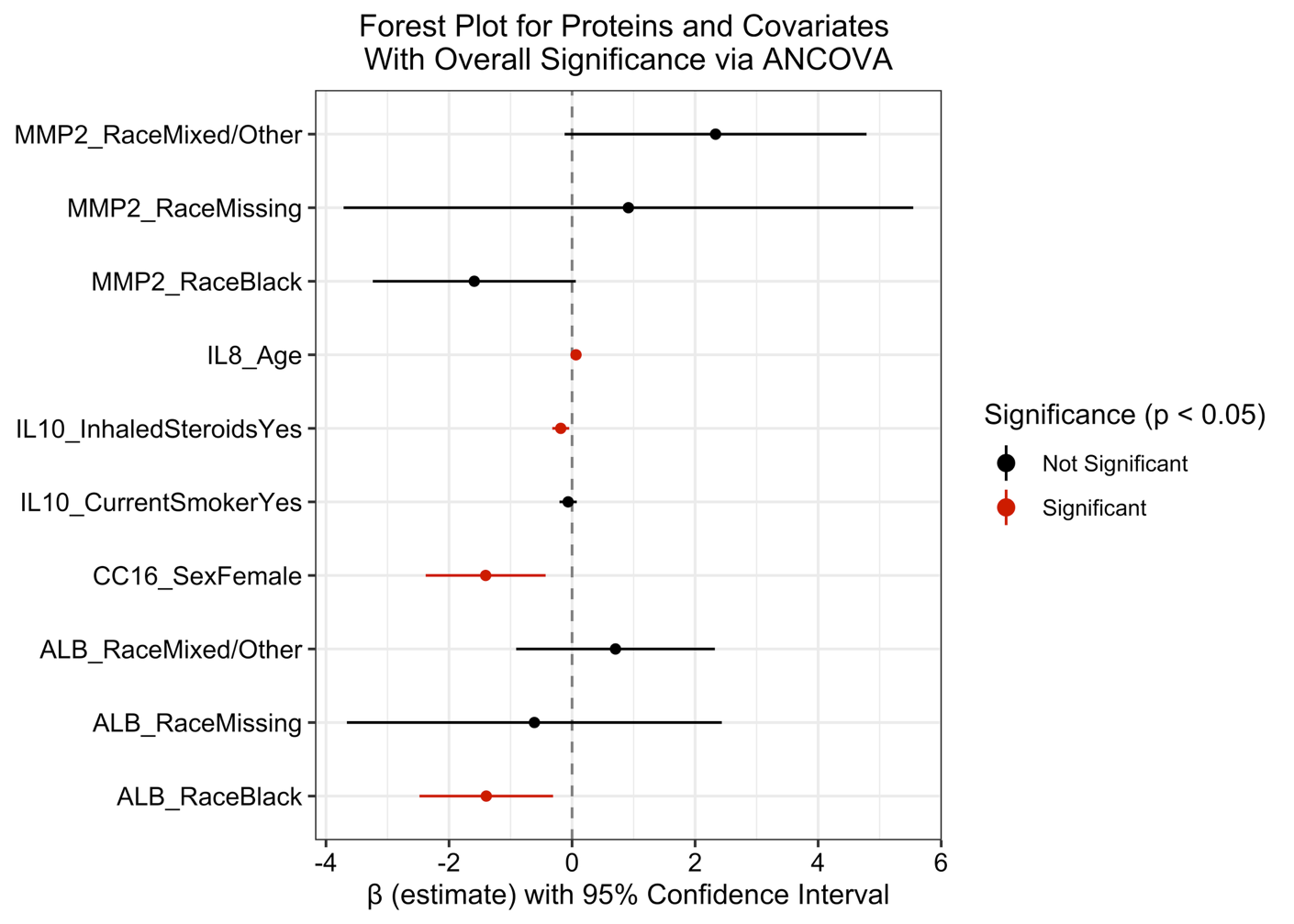** |
| --- |
| **Figure S1. Forest plot for proteins and covariates with overall significance via ANCOVA.** Data are plotted as beta estimate (dot) with 95% confidence intervals (lines). Red highlighting indicates p < 0.05 for that individual term and protein. Full results for all covariates and proteins are available in supplemental table SX. |

| **** |
| --- |
| **Figure S2. Clustering of individual participants with COPD and e-cigarette users across all soluble mediators.** Row-scaled heatmap of log2, batch-adjusted mediator concentrations are shown. The Control group represents a combined group containing COPD study controls and non-smokers/non-vapers from the Hickman et al. e-cigarette study. EC = e-cigarette. |

| **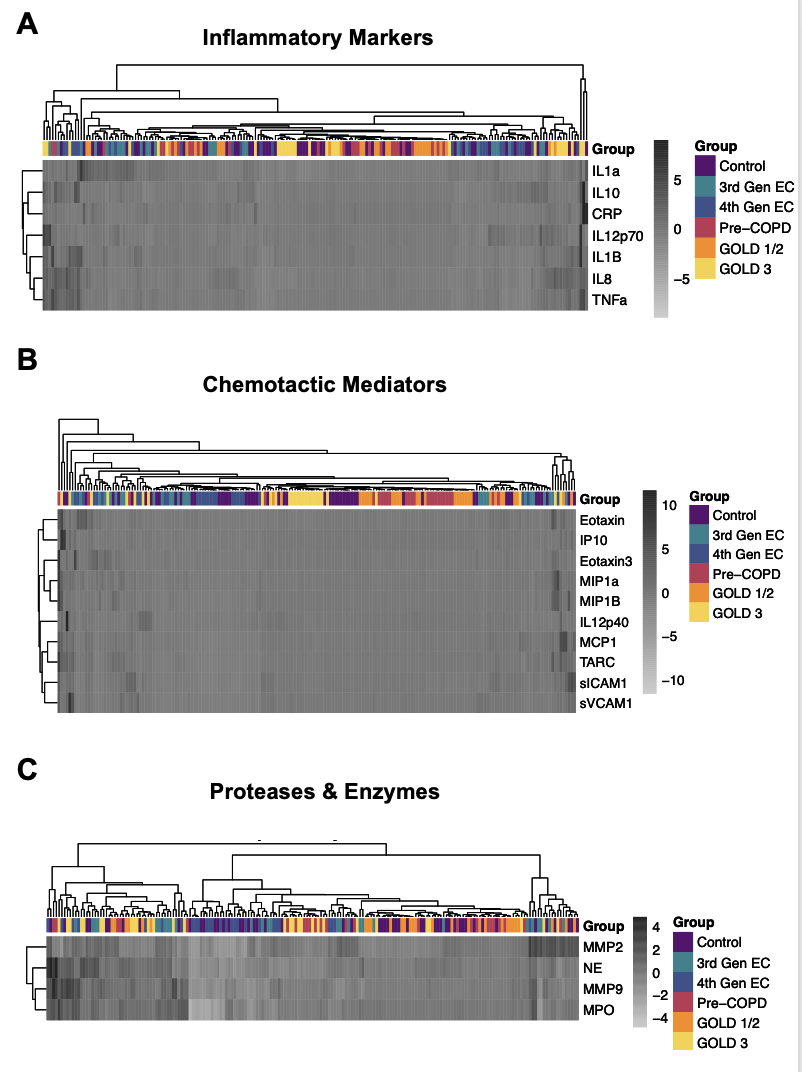** |
| --- |
| **Figure S3. Clustering of individual participants with COPD and e-cigarette users across biological subsets of soluble mediators:** (A) Inflammatory markers, (B) Chemotactic mediators, and (C) Proteases and enzymes. Row-scaled heatmaps of log2, batch-adjusted mediator concentrations are shown. The Control group represents a combined group containing COPD study controls and non-smokers/non-vapers from the Hickman et al. e-cigarette study. EC = e-cigarette. |

| **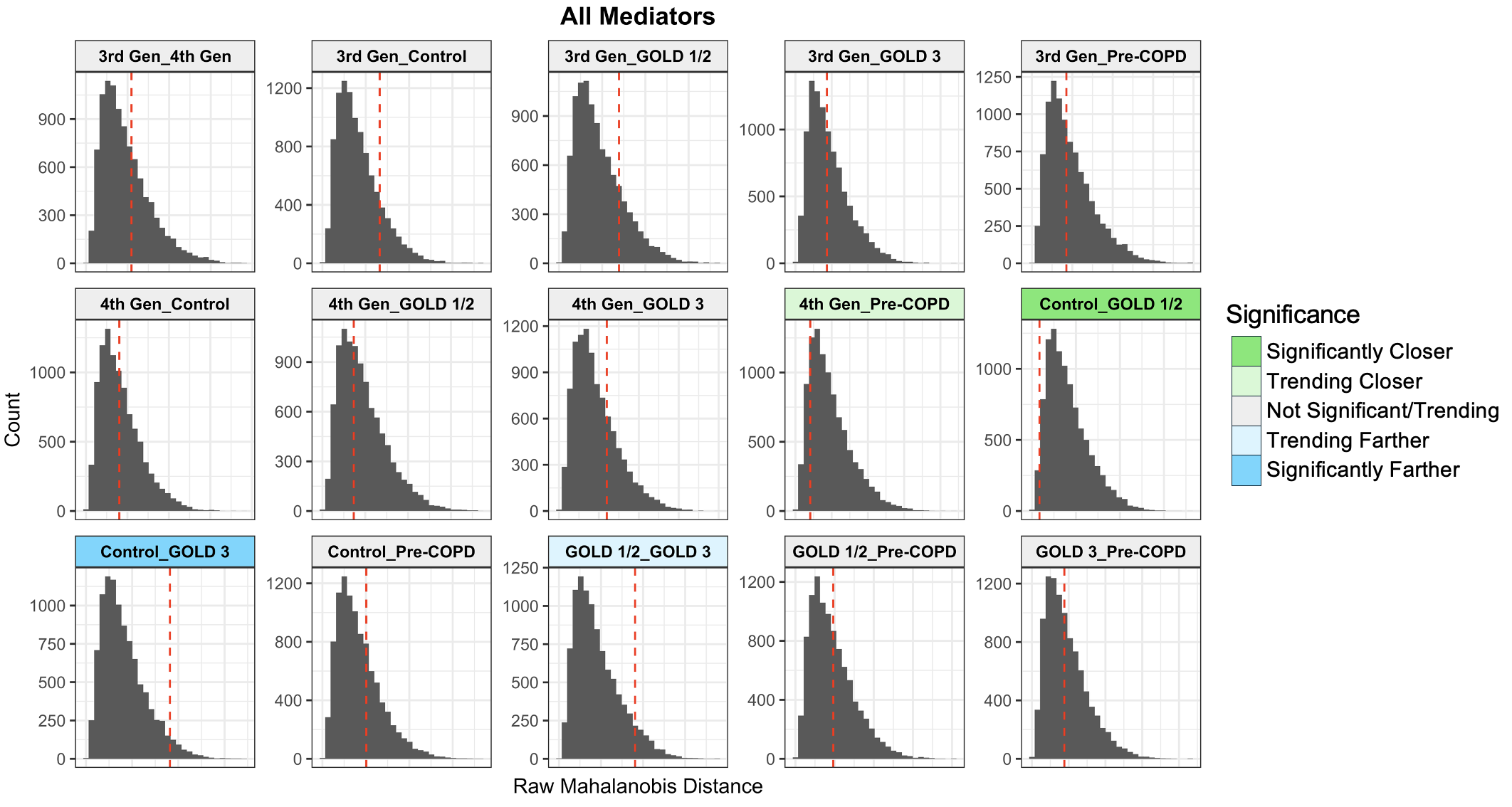** |
| --- |
| **Figure S4. Comparison of actual Mahalanobis distances between groups with random distribution generated by permutation testing for the dataset containing all measured mediators.** Distances < 5^th^ or > 95^th^ percentile were considered significantly closer or farther than expected at random, respectively. Distances between the 5^th^ and 15^th^ or the 85^th^ and 95^th^ percentiles were considered trending towards being closer or farther than expected at random. Distances between the 15^th^ and 85^th^ percentiles were considered not significant or trending. |

| **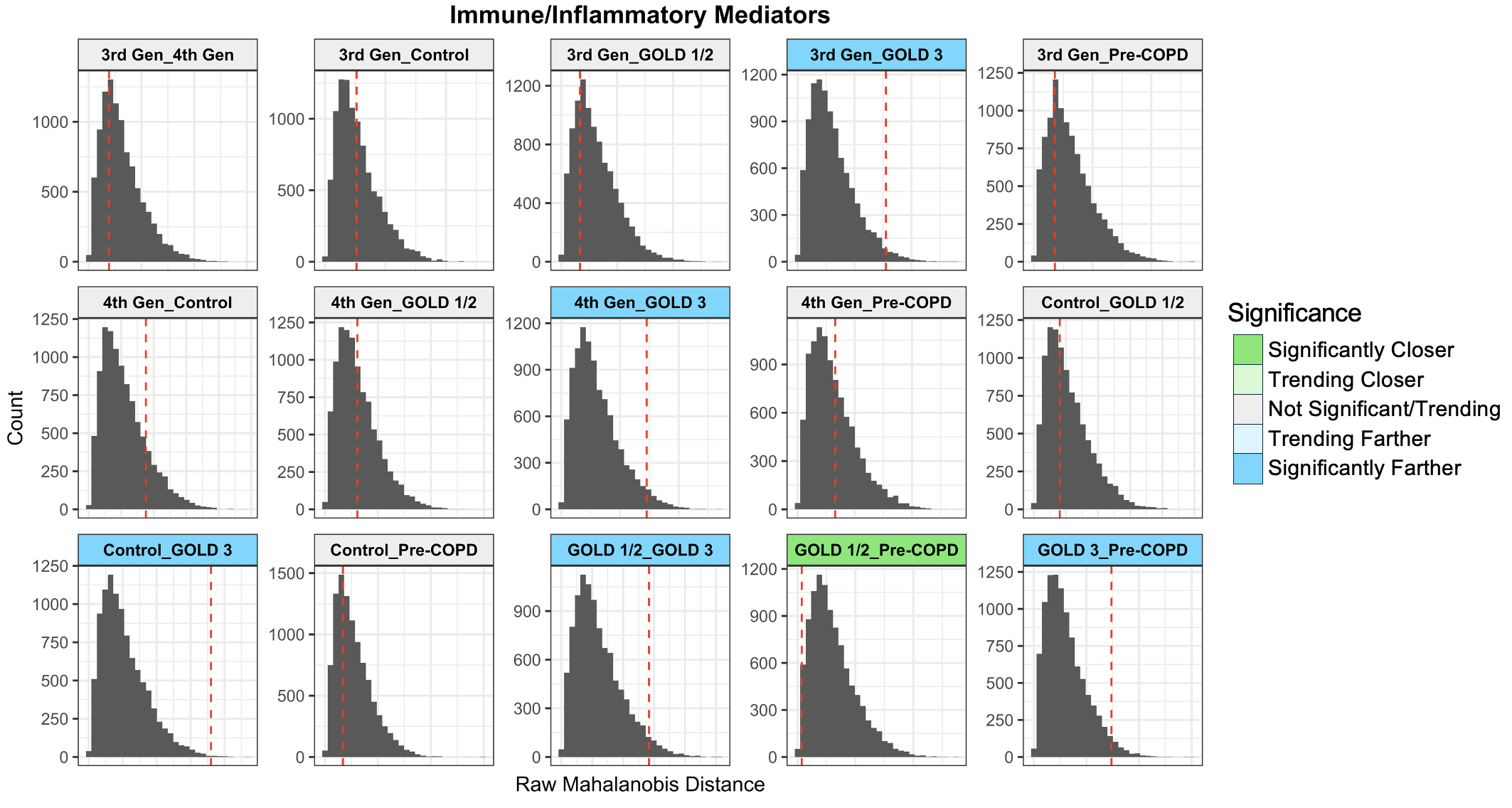** |
| --- |
| **Figure S5. Comparison of actual Mahalanobis distances between groups with random distribution generated by permutation testing for immune/inflammatory mediators.** Distances < 5^th^ or > 95^th^ percentile were considered significantly closer or farther than expected at random, respectively. Distances between the 5^th^ and 15^th^ or the 85^th^ and 95^th^ percentiles were considered trending towards being closer or farther than expected at random. Distances between the 15^th^ and 85^th^ percentiles were considered not significant or trending. |

| **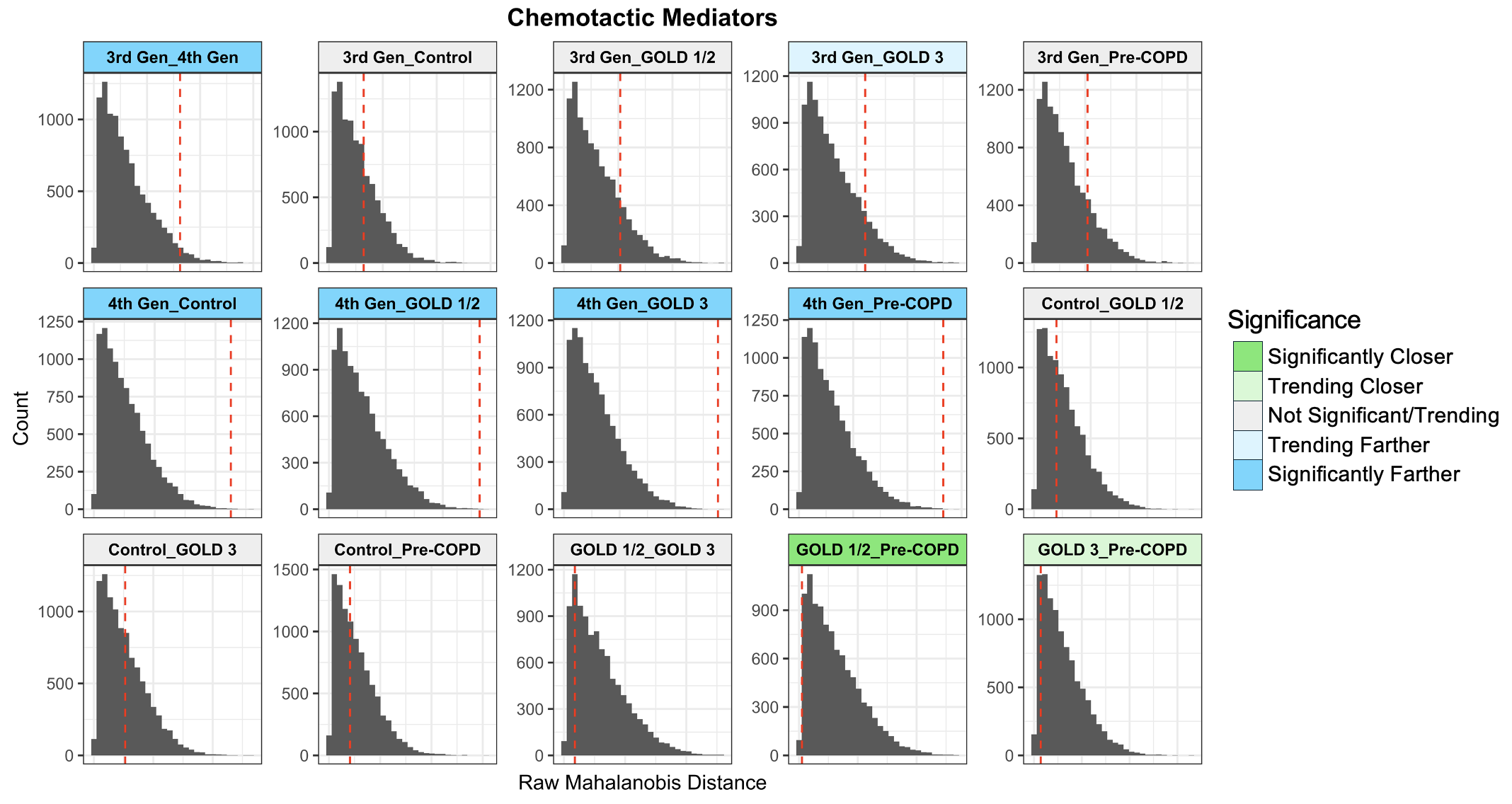** |
| --- |
| **Figure S6. Comparison of actual Mahalanobis distances between groups with random distribution generated by permutation testing for chemotactic mediators.** Distances < 5^th^ or > 95^th^ percentile were considered significantly closer or farther than expected at random, respectively. Distances between the 5^th^ and 15^th^ or the 85^th^ and 95^th^ percentiles were considered trending towards being closer or farther than expected at random. Distances between the 15^th^ and 85^th^ percentiles were considered not significant or trending. |

| **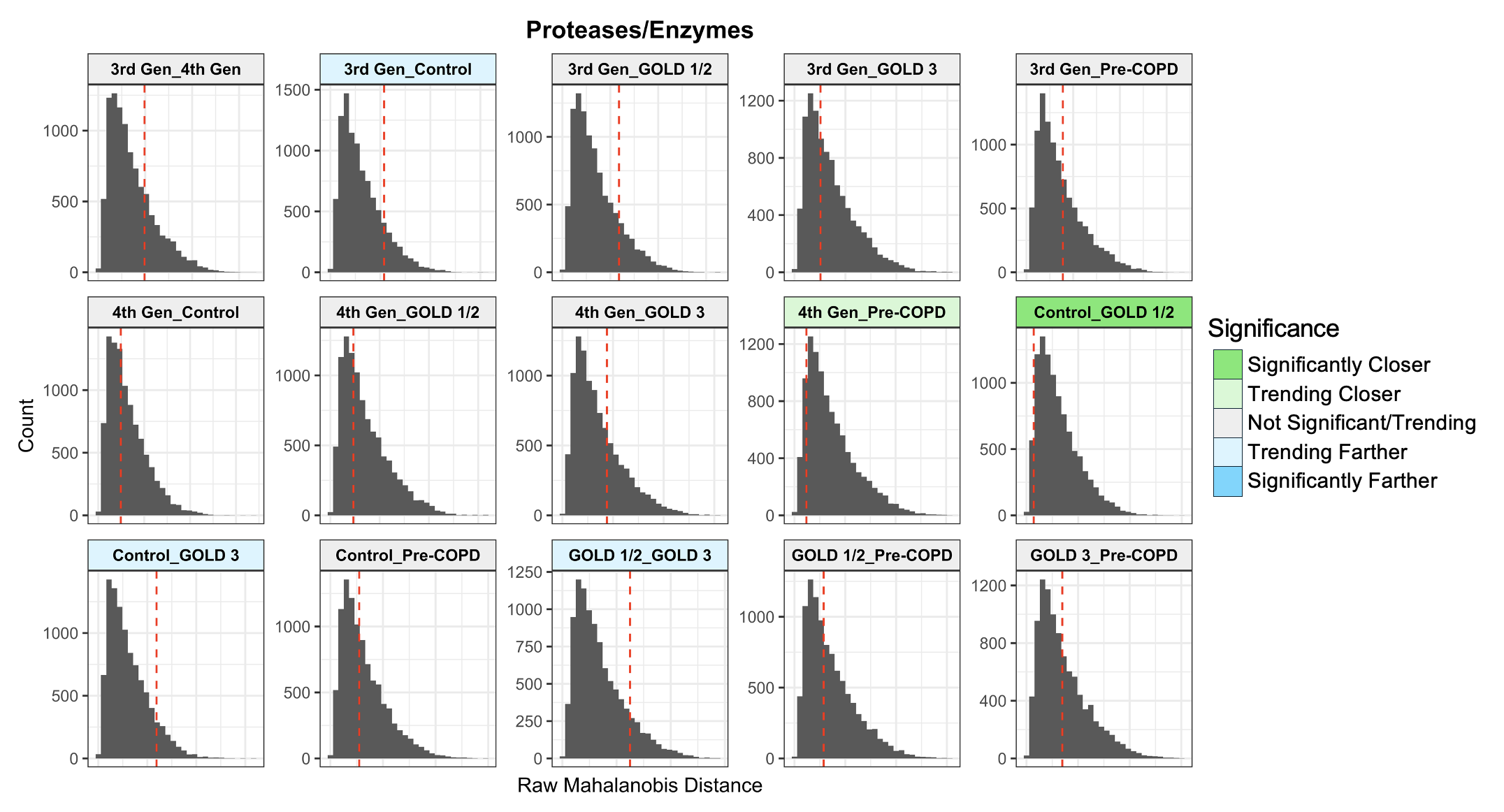** |
| --- |
| **Figure S7. Comparison of actual Mahalanobis distances between groups with random distribution generated by permutation testing for chemotactic mediators.** Distances < 5^th^ or > 95^th^ percentile were considered significantly closer or farther than expected at random, respectively. Distances between the 5^th^ and 15^th^ or the 85^th^ and 95^th^ percentiles were considered trending towards being closer or farther than expected at random. Distances between the 15^th^ and 85^th^ percentiles were considered not significant or trending. |
